# *Rocahepevirus ratti* as an Emerging Cause of Acute Hepatitis Worldwide

**DOI:** 10.3390/microorganisms11122996

**Published:** 2023-12-16

**Authors:** Sara Benavent, Silvia Carlos, Gabriel Reina

**Affiliations:** 1 Microbiology Department, Clínica Universidad de Navarra, 31008 Pamplona, Spain; sbenavent@alumni.unav.es (S.B.); gabi@unav.es (G.R.); 2 Department of Preventive Medicine and Public Health, Universidad de Navarra, 31008 Pamplona, Spain; 3 IdiSNA, Navarra Institute for Health Research, 31008 Pamplona, Spain

**Keywords:** hepatitis E, *Rocahepevirus ratti*, *Orthohepevirus* C, RHEV, HEV-C, rodent, zoonosis

## Abstract

The hepatitis E virus (HEV) is a widespread human infection that causes mainly acute infection and can evolve to a chronic manifestation in immunocompromised individuals. In addition to the common strains of hepatitis E virus (HEV-A), known as *Paslahepevirus balayani*, pathogenic to humans, a genetically highly divergent rat origin hepevirus (RHEV) can cause hepatitis possessing a potential risk of cross-species infection and zoonotic transmission. *Rocahepevirus ratti*, formerly known as *Orthohepevirus* C, is a single-stranded RNA virus, recently reassigned to *Rocahepevirus* genus in the *Hepeviridae* family, including genotypes C1 and C2. RHEV primarily infects rats but has been identified as a rodent zoonotic virus capable of infecting humans through the consumption of contaminated food or water, causing both acute and chronic hepatitis cases in both animals and humans. This review compiles data concluding that 60% (295/489) of RHEV infections are found in Asia, being the continent with the highest zoonotic and transmission potential. Asia not only has the most animal cases but also 16 out of 21 human infections worldwide. Europe follows with 26% (128/489) of RHEV infections in animals, resulting in four human cases out of twenty-one globally. Phylogenetic analysis and genomic sequencing will be employed to gather global data, determine epidemiology, and assess geographical distribution. This information will enhance diagnostic accuracy, pathogenesis understanding, and help prevent cross-species transmission, particularly to humans.

## 1. Introduction

The hepatitis E virus (HEV) is one of the leading causes of hepatitis worldwide. Generally, is presented as an acute infection that improves without treatment after several weeks. Clinical course includes asymptomatic infection, mild-to-moderate liver dysfunction, or fulminant hepatitis. Chronic infections have been reported among immunocompromised individuals like solid organ transplant recipients [1].

**Figure 1:**
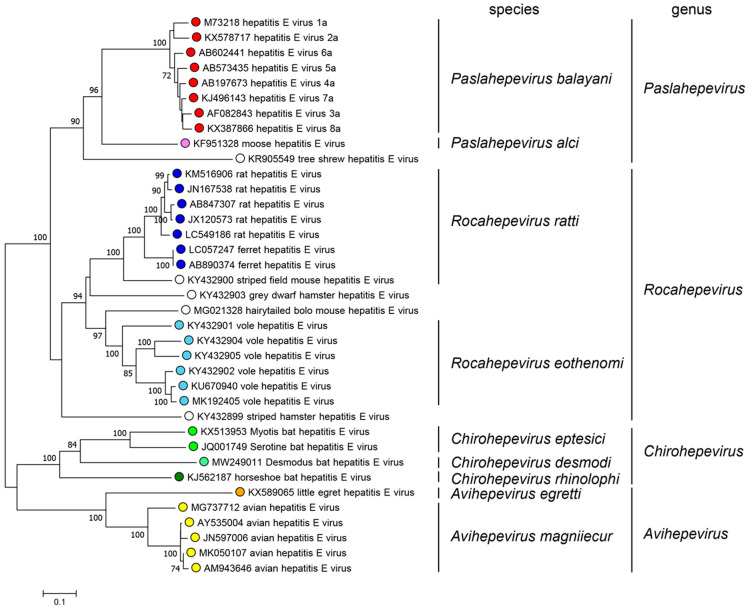
Phylogenetic tree of members of the family *Hepeviridae,* subfamily *Orthohepevirinae* (ORF1 polyprotein residues 1–450) (https://ictv.global/report/chapter/hepeviridae/hepeviridae, accessed on 31 October 2023).

The HEV is a single-stranded RNA virus belonging to the *Hepeviridae* family, which included three genera until 2022: *Orthohepevirus* (infecting mammalian species), *Orthohepevirinae* (infecting unclassified species), and *Piscihepevirus* (infecting fish species). *Orthohepevirus* C (rat hepatitis E virus) also referred as HEV-C, was classified as a member of the *Orthohepevirus* genus within the *Hepeviridae* family, sharing a close genetic relationship with other hepatitis E viruses, including HEV-A, HEV-B, and HEV-D (Figure 1) [2,3].

The current nomenclature for the former *Orthohepevirus* C is *Rocahepevirus ratti* (RHEV), as the International Committee on Taxonomy of Viruses (ICTV) has reclassified the *Orthohepevirus* genus. The other virus pathogen of the *Hepeviridae* family that infects humans is the *Paslahepevirus balayani*, previously known as *Orthohepevirus* A (HEV-A). HEV-A virus exhibits eight genotypes (1–8) and it is primarily transmitted through the fecal-oral route, associated with contaminated water or food, and mainly prevalent in regions with inadequate hygiene practice. While *Paslahepevirus balayani* is recognized as the causative agent of hepatitis E in humans, *Rocahepevirus ratti* represents an emerging zoonotic pathogen primarily infecting rats and other rodent species, this infectious agent can potentially infect humans increasing the concern in terms of public health [4,5].

The genomic sequence analysis of RHEV has revealed new cases involving zoonotic transmission, as rodents are susceptible to genotype 1 (HEV-C1) infection [6]. This virus is primarily associated with rat populations, which can shed the agent in their feces and urine when infected, leading to environmental contamination. The actual transmission route for human infection is unknown, but different risk factors have been proposed, such as exposure to contaminated environments, contact with infected animals, or the consumption of contaminated food or water [7].

Since the RHEV discovery in rats in 2010 in Germany, this virus has been detected in multiple countries across the world in Asia, Europe, and North America [8]. Although the mechanism of RHEV infection in humans is not entirely clear, studies indicate that the virus may cause mild to moderate acute hepatitis, with symptoms including fever, fatigue, nausea, and abdominal pain. Occasionally, there are cases where the RHEV infection may progress to chronic hepatitis or liver cirrhosis, particularly among immunocompromised individuals. Molecular and serological diagnostic techniques are typically employed for identifying RHEV infection [4].

Considering the emerging nature of the *Rocahepevirus ratti* as a human pathogen, there is a pressing need to evaluate the existing literature on this virus, including its phylogenetic and genomic analysis, epidemiology and its existing geographic distribution, pathogenesis, and diagnosis available. The objective of this review is to help to better understand the potential risks associated with RHEV infection and to provide valuable information on devising effective measures for prevention and control of this emerging cross-species infection and zoonotic pathogen.

## 2. Materials and Methods

**Figure 2:**
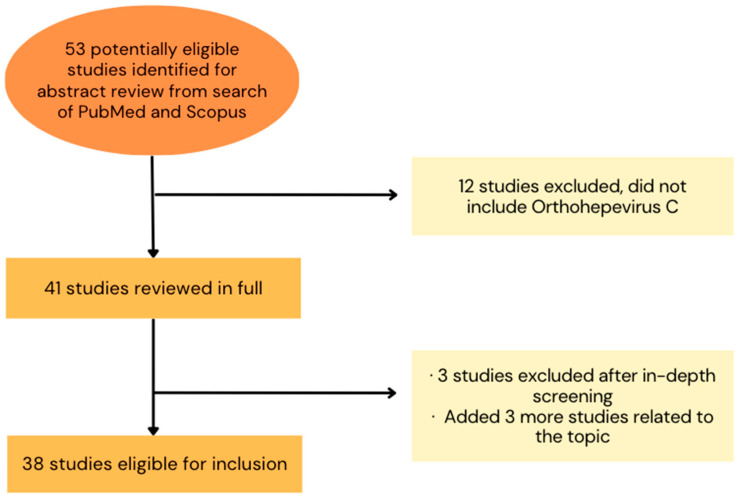
Selection of studies about *Rocahepevirus ratti*, including its epidemiology, 2000–2023. This selection was made for inclusion of studies in the bibliographic review. It was necessary to consider the fact that *Orthohepevirus* C has been renowned as *Rocahepevirus ratti* in recent studies (2022 and 2023).

A bibliographic review was conducted to investigate the detected cases of *Rocahepevirus ratti* as a newly emerging virus worldwide, including its diagnostic data, in both humans and animals. For this purpose, the search was carried out using Pubmed and Scopus with the following keywords: “*Orthohepevirus* C”, “*Rocahepevirus ratti*”, “HEV-C1”, “HEV- C2”, “rodent”, and “zoonosis”. This search aimed to identify the different research developed in human and animal health related to RHEV for a better understanding of this infection. All studies considered were published worldwide in a period spanning from December 2000 to October 2023, in any available language (Figure 2).

To ensure the accuracy and reliability of the research, all available articles were individually evaluated for inclusion. In the study of the animals that contracted the infection, not only those with the complete sequence were considered, data from those with the partial genomic sequence were included after verifying their association with the studied virus’s genomic sequence. Authors of the studies were contacted to clarify any ambiguity or provide additional information. For the genomic sequence, data were collected from studies in which virus RNA was sequenced using Sanger sequencing or NGS. Additionally, public sequence databases like European Nucleotide Archive and GenBank were queried for each case. The working maps were constructed by using a free open source, Mapchart tool (https://www.mapchart.net/world.html, accessed on 31 October 2023) using the collected data from the generated tables. 

The research methodology employed in this review ensures the selection of reliable and accurate data and aims to provide valuable insights into the detected cases and diagnostic information of RHEV globally.

## 3. Phylogenetic Analysis and Genomic Sequence

The *Hepeviridae* family is divided into two subfamilies: *Orthohepevirinae*, divided into four genera: *Paslahepevirus* (former *Orthohepevirus A*) the main cause of hepatitis E, which embraces eight different genotypes affecting a variety of species including humans, pigs, wild boar, deer, rabbits, and camels [7]; *Rocahepevirus* (previous *Orthohepevirus* C) which infects the Rodentia and Soricomorpha order (rats and ferrets), as well as Carnivora; *Chirohepevirus* (old *Orthohepevirus* D) which infects Chiroptera order (bats); and *Avihepevirus* (previous *Orthohepevirus* B) which infects and circulates in the avian order (birds); the second subfamily is *Parahepevirinae* containing one single genus, *Piscihepevirus* [9] (Table 1). RHEV and HEV-A are two highly divergent viruses, their genomes only share 50–60% genomic identity, differing on their transmission and pathogenicity, with RHEV infections being related to less severe disease outcomes [7].

The HEV virus presents eight main genotypes within the *Paslahepevirus genus* [4]. Genotypes 1 and 2 transmission is through the consumption of contaminated water by feces, mainly in developing countries. This situation has triggered zoonotic transmission by these genotypes, due to the contamination of the environment with the pathogen from animal feces or urine. As well as the transmission through the ingestion of raw or undercooked meat of the infected animal host, which predominates in genotypes 3 to 8 [5,6]. Within the RHEV, there are two main genotypes, HEV-C1 and HEV-C2, showing a divergence of 44%. In addition, two extra tentative genotypes (C3 and C4) have been proposed within RHEV species [3].

**Figure 3:**
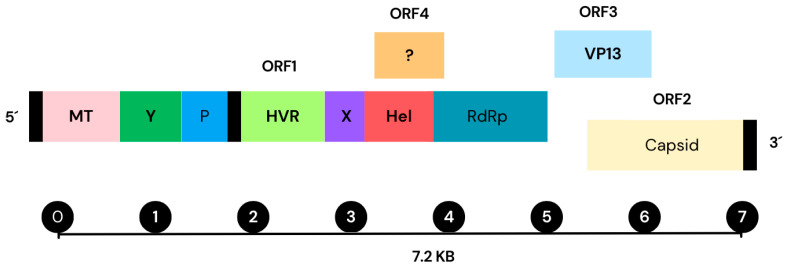
Hepatitis E virus (HEV-A) genome structure, including its open reading frames (ORFs) and proteins. Contains a single-stranded positive-sense RNA molecule approximately 7.2 kb long and is presented in the genome of the virus of HEV-A. It displays a 7-methylguanosine RNA at the 5′ end and poly-A at the 3′ terminus. All HEV strains contain three open reading frames (ORFs): ORF1, ORF2, and ORF3 (an additional ORF4(?) region is found which functionality is not totally understood). ORF1 produces non-structural polyproteins for viral replication and transcription such as (Met), Y-domain, (PCP), (HVR), (Hel), and (RdRp). ORF2 encodes the capsid protein, while ORF3 encodes a multifunctional phosphoprotein, known as VP13. These proteins from ORF2 and ORF3 exhibit partial overlap and are translated from a subgenomic RNA with 2.2 kb in length. Additionally, ORF4 gives rise to an internal ribosome entry site-like protein (IRES) to respond to the stress generated in the endoplasmic reticulum (ER). ORF4 is an enhancer of viral replication. (GenBank accession number AF444002.1) [10,13,14].

According to the genomic sequence analysis, the HEV-A virus possesses a single- stranded RNA genome positive-sense approximately 7.2 kb in length. The genome encodes three open reading frames (ORFs) which participate in the viral replication and transcription [9,10] (Figure 3). ORF1 contains a large polyprotein that is processed into several non- structural proteins, including RNA-dependent RNA polymerase (RdRp), helicase, and protease. ORF2 codifies a protein for the viral capsid, while ORF3 produces a minor protein responsible for the virion assembly and release. The genomic structure of HEV-A resembles other hepeviruses, like the RHEV [11]. Recent studies have identified a new open reading frame (ORF4), located within the ORF1 region of the HEV-A genome. This ORF4 encodes a 20 kDa protein with 139–158 amino acids span. Despite its discovery, the role of the ORF4 in the HEV-A or RHEV viral replication and pathogenesis remain unclear [12]. Investigating the ORFs of the RHEV virus is crucial to understand the genetic structure of the virus, the pathogenesis, replication, transcription and translation mechanisms, and how they adapt into the host to develop more effective strategies against this pathogen [11]. Additionally, despite a number of studies on the genomics of HEV-A and RHEV, there is still a limited knowledge on the evolutionary history and functions of the different regions. Comparing the genomic sequence of RHEV with other members of the *Hepeviridae* family, as well as to other related viruses, such as the *Flaviviridae* family, can provide valuable insights into the evolutionary history of the virus and how it has evolved to infect different host species [2]. Such comparisons can identify regions of similarity in the different strains that can be targeted to develop antiviral drugs or utilized as diagnostic markers for detection. Conducting phylogenetic analysis and genomic sequencing of RHEV is crucial for understanding how the virus evolves.

## 4. Epidemiology and Geographical Distribution

### 4.1. Human Infections Associated with Rocahepevirus ratti

At this moment, up to 21 cases of HEV-C1 infection have been reported in humans through RNA detection in different samples. This data includes sixteen patients in Hong Kong, three patients in Spain, one patient in Central Africa, and one patient in France. RHEV initial detection occurred in rats from Germany and Vietnam [3], but no human cases have been found in Germany [15] or Hungary [16] after investigation. In Spain, a comprehensive study conducted by Rivero-Juarez et al. revealed the presence of *Rocahepevirus ratti* infection in three human patients. Two of these cases were located in the southern region, while one was identified in the northern part of Spain (Table 2).

In China, particularly in Hong Kong, three studies have been carried out to investigate the role of *Rocahepevirus ratti*. The most recent of these studies was published in 2022 and focused on a cohort of 53 patients from the Microbiology Department of Queen Mary Hospital. This study was conducted between 1 August 2019 and 31 December 2020, and the results indicated a total of eight cases with positive RNA HEV-C1 [17]. This study was a continuation of a prior investigation, which analyzed 2201 sera from patients with liver function abnormalities between 1 January 2017 and 31 July 2019 (group 1), and 659 cases in a second group, composed of transplant recipients and patients with solid organ neoplasms, hematologic neoplasms, autoimmune disorders, and other immunosuppressive conditions between 1 January 2019 and 30 June 2019 (group 2). The previous study identified six cases of HEV-C1 RNA positivity in group 1 and one case in group 2, leading to a total of seven cases [18]. Notably, the first confirmed human infection caused by RHEV, originating from rats, was identified in Hong Kong in 2017. The study examined a patient who was Anti-HEV IgG positive and had borderline Anti-HEV IgM results. This patient was found to have persistent Hepatitis E after receiving a liver transplant. For this research, a total of 518 solid organ transplant recipients, including kidney, liver, lung, and heart transplants, were evaluated. These recipients exhibited persistent hepatitis, defined as an elevation of alanine aminotransferase (ALT) greater than 1.5 times the upper limit of the reference level for a continuous period of more than six weeks, from 1 January 2014, or the date of transplant (whichever was later) until 31 December 2017 [19] (Table 3).

Another reported case of RHEV infection in humans involved a Canadian citizen who worked for the United Nations (UN) and was admitted to a hospital returning home in 2017 with severe acute hepatitis. Before his hospitalization, he had worked at various UN facilities in the Democratic Republic of Congo and Gabon from January to 12 March 2017. The exact origin of his infection remains uncertain (Gabon or DRC). Nevertheless, this case is noteworthy as the first and only case with origins from the African continent, where hepatitis E is more prevalent than in other continents. In this case, the infection is believed to have been transmitted through zoonosis, as it is RHEV [20] (Table 3).

Recent studies have been conducted, revealing also the transmission of RHEV to humans in Europe. A recent study, conducted in Spain, examined 267 cases and evaluated two cohorts: one comprising 169 patients without HEV infection, and the other consisting of 98 patients diagnosed of HEV infection (either HEV RNA or HEV-IgM positive). In the first cohort, two cases of HEV-C1 RNA-positive samples were detected, whereas one case was found in the second cohort (HEV-IgM(+)). Therefore, the first RHEV recorded cases in Europe were these three human infections identified in Spain [4]. A fourth European case was discovered in France thanks to metagenomics testing, revealing the presence of RHEV in liver/blood samples of a male who developed cirrhosis after resolving HBV infection (loss of HBsAg and non detectable DNA). This patient of Indian origin underwent renal transplantation in 2006 and despite successful treatment for hepatitis B virus (HBV) reactivation in 2008, he developed cirrhosis in 2016, leading to a double kidney-liver transplantation. Post-transplantation, the patient experienced unexplained liver abnormalities. In 2022, a pan-pathogen non-specific metagenomics method revealed the presence of RHEV in liver samples since 2017, indicating a persistent infection despite undetectable HBV DNA, so the patient’s cirrhosis was attributed to RHEV [22] (Table 3).

It is worth noting that some studies have been conducted where no positive cases were detected. A study conducted in Germany involved the evaluation of 200 patients who tested positive or borderline positive for HEV-IgM but negative for HEV RNA. This study did not yield any RHEV positive cases [15]. Similarly, in France, 224 individuals at risk of developing chronic HEV infection were assessed, and none of them tested positive for RHEV RNA [21]. Similarly, to a group of 163 individuals in Hungary with HEV-IgM(+) marker, where no RHEV was found [16].

There is scarce information regarding RHEV seroprevalence as antibody detection is not highly specific, despite amino acid homology of only 55 [8,23]. HEV and RHEV antibodies may cross-react so once specific tools could be developed, it will be possible to get a better knowledge of the circulation of RHEV as subclinical infections may be frequent. In a first attempt to study RHEV seroprevalence in Germany, sera of healthy forestry workers showed a stronger response to HEV-C1 antigen than to HEV-3 antigen [24]. A similar pattern of greater immune response of IgG and IgM against HEV-C1 antigen was observed in a number of patients in Vietnam after febrile illness [25]; and a third study using the immunodominant E2 peptide sequence of HEV-C1 established the antigenic basis for designing the first validated EIA/immunoblots in human populations [26]. Therefore, this method described in Hong Kong has allowed to estimate RHEV seroprevalence of 0,92%, indicating endemic exposure of the population in this region [27].

### 4.2. Animal Infections Associated with Rocahepevirus ratti

Regarding the discovery and dissemination of RHEV on a global scale, including regions such as Europe, Asia, America, and Africa, the Table 4 provides comprehensive data concerning the prevalence of cases, categorized by continents and individual countries.

Until 2023, a cumulative total of 489 infections have been reported in different animals (mainly rodents), notably revealing Asia as the continent with the highest incidence of infected animals, accounting for a total of 295 documented instances. China emerges as the country with the highest reported cases within the Asian region. Europe, on the other hand, stands as the second-highest affected continent, with a combined total of 128 confirmed cases distributed across 15 different countries. In America, the presence of RHEV has only been detected in the United States, Canada, and Brazil, providing a combined total of 65 reported infections. In Africa, a solitary case has been identified, specifically in Kenya (Table 4).

**Figure 4:**
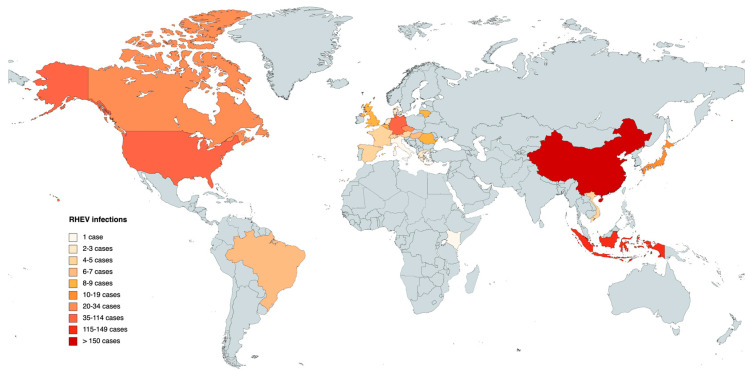
Map of animal infections caused by *Rocahepevirus ratti* in different countries. The darker the color, the greater the number of infections.

The global map in Figure 4 corresponds to the data presented in Table 4, offering a comprehensive review of the results pertaining the different countries. The data are differentiated by RHEV animal infections, and the colored regions on the map represent the locations where detected animal infections have been reported.

The first identification of hepevirus associated with rodents was made back to 2010 when a nested broad-spectrum reverse transcription polymerase chain reaction (RT-PCR) was employed in Germany. Subsequent genome sequencing revealed substantial genetic disparities between this virus and the human HEV strain. After thorough examination, it was classified as *Rocahepevirus ratti* and assigned the genotype HEV-C1. Following its initial discovery in Germany, RHEV was subsequently found in a wide range of animal species (Table 5).

Notably, the Rodentia genus exhibited the highest prevalence of detected infections, encompassing wild Norway rats (*Rattus norvegicus*) across 22 countries, black rats (*Rattus rattus*) in 16 countries, which included 12 European nations (Austria, Belgium, Czech Republic, Denmark, France, Germany, Greece, Hungary, Italy, Lithuania, Spain, and Switzerland), as well as Tanezumi rats (*Rattus tanezumi*) in Vietnam, where infections were observed.

According to these findings the widespread presence of HEV-C1 among rat populations across the four continents, Asia, Europe, America, and Africa. Furthermore, infections were identified in the *Cricetidae* family, including the Asian musk shrew (*Suncus murinus*). HEV-C2 genomes were also detected in ferrets and minks.

Beyond these classifications, there have been reports of unclassified rodent RHEV strains, which include Chevrier’s field mouse (*Apodemus chevrieri*), Pere David’s mole (*Eothenomys melanogaster*), hairy-tailed vole mouse (*Necromys lasiurus*), delicate vole mouse (*Calomys tener*), among others [7]. It is remarkable that RHEV infections are not limited to species within the Rodentia order; there have also been documented cases of *Rocahepevirus ratti* in the Soricomorpha order in two countries, China and Kenya, as well as in members of the Carnivora order, comprising six different species, found in countries such as Denmark, the Netherlands, the United States, Japan, China, Germany, and Spain. Additionally, two species from the order Falconiformes, the Common Kestrel (*Falco tinnunculus*) and the Red-footed Falcon (*Falco vespertinus*), were also identified as infected by RHEV in Hungary (Table 5).

### 4.3. Investigation of Rocahepevirus ratti in Wastewater Systems

Sewage surveillance is recognized as a powerful tool to gather information on the epidemiology of infectious diseases in the served population. The presence of RHEV has been explored in sewage treatment plants at different locations in Sweden, Italy, and Spain, allowing to get a better view of its epidemiology.

In Gothenburg (Sweden), a comprehensive study monitored influent and effluent wastewater for enteric viruses by qPCR, at the Rya treatment plant over a year. Seasonal variations in viral concentrations were observed in incoming wastewater, correlating with the number of diagnosed patients. Different HEV strains previously identified in drinking water, including two novel strains similar to those infecting rats and humans, were identified in the effluents [28]

Another study in Abruzzo (Italy), detected viral RNA of RHEV in 43.9% of sewage collected from 14 wastewater treatment plants, by using broadly reactive primers for hepevirus. These strains exhibited genetic variability and a clear geographic and wastewater treatment plant-related pattern. This study suggested that RHEV was a significant component of wastewater microbiota in the region [29]

Finally, a longitudinal study was carried out in Cordoba (southern Spain) from 2021 to 2023 using samples from patients with acute hepatitis, specimens from rodents, and wastewater to evaluate the correlation with human cases. The results showed that while RHEV was detected in almost all wastewater samples, there was no correlation between clinical cases and wastewater detection for both HEV and RHEV [30]

These studies highlight the importance of monitoring wastewater for RHEV. Untreated wastewater collects viruses excreted by both humans and synanthropic animals, including rodents, thereby providing a comprehensive overview of the viral strains circulating. Despite the reduction in viral concentrations during wastewater treatment, the presence of novel strains concerns the potential public health impact. Further research in this area is essential to understand the epidemiology and impact of RHEV infections on human health.

**Table 5:** Summary of animal infections associated with rat origin HEV-C1 virus (*Rocahepevirus ratti*).

Order/Family	Species	Type of Detection	Year and Location	Type of HEV	GenBank Acc. Nº	Type of Genomic Sequence	Ref.
Rodentia/ Muridae	Norway rat (*Rattus norvegicus*)	2 HEV IgM+/134 sera; 1 RT-PCR+	2003 (Los Angeles; USA)	HEV-C1	JF516246	Complete/Partial	[31]
	Norway rat (*Rattus norvegicus*), Black rat (*Rattus rattus*)	63 RT-PCR+/508 liver samples	2005–2016 (12 European countries)	HEV-C1 (49)	KX774641–KX774673	Complete/Partial	[32]
	Norway rat (*Rattus norvegicus*)	14 RT-PCR+/101 liver samples	2008–2010 (Hamburg, Berlin, Stuttgart, Esslingen; Germany)	HEV-C1	JN167530–JN167538	Complete/Partial	[33]
	Norway rat (*Rattus norvegicus*)	2 RT-PCR+/6 liver samples	July 2009 (Hamburg; Germany)	HEV-C1	GU345042, GU345043	Complete	[34]
	Norway rat (*Rattus norvegicus*), Tanezumi rat (*Rattus tanezumi*)	5 HEV IgM+/139 sera; 1 RT-PCR+	2011 (Haiphong and Hanoi; Vietnam)	HEV-C1	JN040433 (=JX120573)	Complete/Partial	[35]
	Norway rat (*Rattus norvegicus*) Greater bandicoot rat (*Bandicota indica*), *Rattus flavipectus, Rattus rattoides losea*	59 HEV IgM+/713 sera; 12 RT-PCR+	December 2011–September 2012 (Zhanjiang, China)	HEV-C1	KC465998–KC465999	Complete/Partial	[36]
	Norway rat (*Rattus norvegicus*), Black rat (*Rattus rattus*)	35 RT-PCR+/446 liver samples	2012 (15 states; USA)	HEV-3 (34) HEV-C1 (1)	JQ898480–JQ898514	Complete	[37]
	Norway rat (*Rattus norvegicus*)	8 RT-PCR+/61 liver samples	2014–2016 (Great Britain)	HEV-C1	MK770165–MK770171	Complete	[38]
	Norway rat (*Rattus norvegicus*)	7 RT-PCR+/159 rectal swabs	2018–2019 (Hong-Kong, China)	HEV-C1	MN450855, MN450859– MN450864	Complete	[18]
	Norway rat (*Rattus norvegicus*)	1 RT-PCR+/10 fecal samples	2017–2018 (Budapest; Hungary)	HEV-C1	MT847624	Complete	[3]
	Norway rat (*Rattus norvegicus*)	21 RT-PCR+/372 liver samples	2018–2021 (Ontario Canada)	HEV-C1	OQ617169–OQ617185	Partial	[39]
	Norway rat (*Rattus norvegicus*)	9 RT-PCR+/69 liver samples	2016–2023 (Eastern Romania)	HEV-C1	OQ601523–OQ601531	Partial	[40]
Rodentia/ Muridae (continue)	Black rat (*Rattus rattus*)	17 RT-PCR+/116 sera	August 2011–February 2012 (Lambok Island; Indonesia)	HEV-C1	AB725884–AB725900	Complete	[41]
	Black rat (*Rattus rattus*)	99 RT-PCR+/369 sera	September–October 2012 (Solo, Indonesia)	HEV-C1	AB847305–AB847406	Complete/Partial	[42]
	Black rat (*Rattus rattus*)	2 RT-PCR+/242 sera	2014–2016 (Bali and Sumbawa, Indonesia)	HEV-C1	LC225388–LC225389	Complete	[43]
	Black (*Rattus rattus*), Norway rats (Rattus norvegicus)	9 RT-qPCR+/109 liver and chest fluid samples	2014–2017 (Lithuania)	HEV-C1	MH400712–MH400717	Complete	[44]
	Black (*Rattus rattus*), Norway rats (*Rattus norvegicus)*	5 HEV IgG+/20 fecal samples/7 tissue samples/428 sera	2014–2017 (Japan)	HEV-C1	LC573546	Complete	[45]
	Chevrier’s field mouse (*Apodemus chevrieri*)	59 RT-PCR+/202 sera	2013–2015 (Lijiang, China)	HEV-C	MG020022–MG020025	Complete	[6]
	Striped field mouse (*Apodemus agrarius*)	2 RT-PCR+/2 sera	2014 (Jilin; China)	HEV-C	KY432900	Complete	[46]
Rodentia/ Cricetidae	Hairy-tailed mouse (*Necromys lasiurus*), Delicate vesper mouse (*Calomys tener*)	4 RT-PCR+/109 sera 3 RT-PCR+/252 sera	2008–2013 (Brazil)	HEV-C	MG021328	Partial	[47]
	Bank vole (*Myodes glareolus*)	1 RT-PCR+/1206 sera	2009–2013 (Germany)	HEV-C	MK192412	Partial	[48]
	Common vole (*Microtus arvalis*)	13 RT-PCR+/646 sera	2009–2013 (Germany, Czech Republic) and 2016 (Hungary)	HEV-C	MK192405-MK192409 MK192413-MK192420	Complete/Partial	[48]
	Pere David’s vole (*Eothenomys melanogaster)*	4 RT-PCR+/55 sera	2013–2015 (Lijiang, China)	HEV-C	MG020022–MG020025	Complete	[6]
	Inez’s red-backed vole (*Eothenomys inez*)	2 RT-PCR+/23 sera	2014 (Shanxi; China)	HEV-C	KY432904	Complete	[46]
	Chinese striped hamster (*Cricetulus barabensis*)	5 RT-PCR+/60 sera	2014 (Hebei; China)	HEV-C	KY432899	Complete	[46]
	Narrow-headed vole (*Microtus gregalis*)	4 RT-PCR+/44 sera	2015 (Xinjiang; China)	HEV-C	KY432902	Complete	[46]
	Grey red-backed vole (*Myodes rufocanus*)	3 RT-PCR+/23 sera	2015 (Heilongjiang; China)	HEV-C	KY432901	Complete	[46]
	Gray Dwarf Hamster (*Cricetulus migratorius*)	1 RT-PCR+/15 sera	2015 (Xinjiang; China)	HEV-C	KY432903	Complete	[46]
Soricomorpha/ Soricidae	Asian musk shrew (*Suncus murinus*)	12 HEV IgM+/260 sera; 5 RT-PCR+	December 2011–September 2012 (Zhanjiang; China)	HEV-C1	KC465990–KC466001	Complete/Partial	[49]
	Oliver’s shrew (*Crocidura olivieri*)	1 RT-PCR+/5 sera	2014 (Kenya)	HEV-C	NA	Partial	[50]
Carnivora/ Mustelidae	Western Ferrets (*Mustela putorius*) American mink (*Neovison vison*)	2 RT-PCR+/63 fecal samples	2008–2010 (Denmark) 2010 (Netherlands), 2013 (USA), 2009–2013 (Japan), 2016 (China)	HEV-C2	AB890375-AB890379 LC177789-LC177791 JN998606-JN998607	Complete/Partial	[51]
	Syrian brown bear (*Ursus arctos syriacus*)	2 NBS-RT-PCR+/22 sera	20011–2016 (Germany)	HEV-C1	MF480313	Partial	[52]
	Red Foxes (*Vulpes vulpes*)	1 RT-PCR+/26 fecal samples	2020 (Hungary)	HEV-C2	MN906015	Complete	[53]
	Dog (*Canis familiaris*) Cat (*Felis catus*)	2 HEV Ig+/296 sera; 1 cat/1 dog	2020 (Spain)	HEV-C1	NA	Partial	[54]
Farconiformes/ Falconidae	Common kestrel (*Falco tinnunculus*) and Red-footed falcon (*Falco vespertinus*)	2 RT-PCR+/18 fecal samples 1 RT-PCR+/7 fecal samples	2014 (Hungary)	HEV-C	KU670940	Complete/Partial	[55]

Abbreviations: +: positive; NA: Not Available.

## 5. Pathogenesis and Diagnosis

*Rocahepevirus ratti,* like *Paslahepevirus balayani*, can cause acute hepatitis, chronic hepatitis, and subclinical infection. HEV-C1 infections are self-limiting and less severe than HEV-A infections, characterized by lower mean peak ALT and bilirubin levels in those with intact immunity. In general, the criteria for inclusion in these investigations to identify RHEV infection include clinical and biological manifestations consistent with acute or chronic hepatitis, as well as an ALT level significantly greater than the upper limit of normal. Infections in immunocompromised individuals, on the other hand, are challenging, with 50% of HEV-C1 infections progressing to persistence, according to research by S. Sridhar et al. [18]. This may be due to the fact that immunocompromised people might be more vulnerable to HEV-C1 infections. Not only was the viral pathogenicity examined, but meningoencephalitis was detected as a sequela in an immunocompromised patient after the hepatitis had entirely cured. Therefore, the correlation between RHEV infection and neurological symptoms must be studied. Subclinical HEV-C1 infection, without changes in hepatic function tests or clinical symptoms, is concerning over the safety of blood transfusions because the virus can be transmitted through contaminated blood samples due to the high viral load of HEV-C1 in plasma. RHEV’s viral tropism is oriented towards the liver, with a special affinity for liver cells. Virus replication occurs there, leading to liver damage and the release of liver enzymes into the blood, resulting in acute and chronic hepatitis in humans and animals. Recognizing the pathogenicity is crucial for the development of therapies for the prevention and treatment of hepatitis caused by this virus [18].

Ferrets and rats have been proposed as candidate animal models to study RHEV pathogenesis. Ferrets exhibit three patterns of infection: subclinical infection, acute hepatitis, and persistent infection [56]. Induced infections of RHEV on immunosuppressed rats have shown the effect of high-dose immunosuppression to induce chronic hepatitis and viral load suppression was observed with ribavirin treatment [57].

Molecular biology techniques have facilitated the identification of RHEV RNA in liver, feces, and blood samples obtained from various animal and human species. Such techniques include RT-PCR amplification, Sanger DNA sequencing, and metagenomic tests involving next generation sequencing. The combination of these methods, along with detailed phylogenetic and sequence evaluations, have allowed sequencing of RHEV genomes [4]. The first human RHEV infection was detected in a patient with chronic hepatitis, and this case was validated by RT-PCR and sequencing of plasma, stool, saliva, and liver tissue samples.

Currently, there are no commercially available serological methods to identify antibodies against RHEV or differentiate them from antibodies against HEV-A. Due to the risk of false negatives, the diagnosis cannot be based on serological tests alone. In addition, HEV-1 to HEV-4 have not been shown to provide protection against RHEV infection. However, a new specific immunoassay for RHEV antibody detection has been developed based on HEV-C1 p241 peptide and validated in solid organ recipients and immunocompetent individuals. This tool allowed to estimate a RHEV seroprevalence of 0.92%, indicating endemic exposure in Hong Kong [27]. This peptide (HEV-C1 p241 antigen) has been shown to be an immunogenic vaccine candidate against HEV-C1 [26].

The most accurate method for detecting *Rocahepevirus ratti* infection is the RT-PCR method by which viral genomic RNA can be identified. The sensitivity of the method depends on the design and selection of specific primers. The molecular diagnosis of HEV-A and RHEV may be challenging as several assays have shown low sensitivity for endemic genotypes in Europe and the number of genotypes/subtypes described is increasing, therefore, without an evaluation, their sensitivity in this context is unknown. Primers used to detect human HEV may not be able to recognize the RHEV genome in patient samples, limiting the sensitivity of PCR, and only the use of specific primers for RHEV has allowed its detection in different samples. It is important to note that the genetic diversity of HEV-C1 is still unclear, which could also limit the sensitivity of the techniques [19]. A recent study conducted by Lopez-Lopez et al. showed that the use of two parallel PCR assays (using ORF1 and ORF3) targeting different regions of the viral genome leads to significant improvement in the molecular diagnosis of RHEV, which can be a useful tool to map the transmission routes of HEV-C1 and hence to prevent human infections [58].

## 6. Conclusions

An increasing number of cases of animals and humans infected with *Rocahepevirus ratti* are being detected. Some species of rats have been confirmed as natural reservoirs of RHEV, and they are considered a source of zoonotic infection. It is likely that more cases will be discovered as detection techniques improve. Understanding the virus evolution and diffusion is essential for the development of successful prevention and control measures, treatments, and vaccines. The information obtained from the phylogenetic analysis and genomic sequencing will help researchers track the development of new strains, contributing to epidemiological distribution maps. In addition, this information can also be used to develop diagnostic tests to accurately detect the virus in collected samples and evolve in the discovery of a more precise pathogenesis. These tests are needed to assist healthcare professionals in diagnosing RHEV infections and preventing the spread of the virus. This is a public health concern because it is a new pathogen that can be transmitted to other species and even to humans. Further research is needed to determine the exact route of transmission of the virus to humans, as it remains unknown.

## Figures and Tables

**Table 1:** Classification of the *Hepeviridae* family and host range [2,9].

Family	Subfamily	Genus/Species	Genotype	Host Range/Reservoir
*Hepeviridae*	*Orthohepevininae*	*Paslahepevirus balayani*	HEV-1	human
			HEV-2	human
			HEV-3	human, pig, wild boar, deer, rabbit, camel
			HEV-4	human, pig, wild boar,
			HEV-5	wild boar
			HEV-6	wild boar
			HEV-7	camel
			HEV-8	camel
		*Paslahepevirus alci*		moose
		*Avihepevirus*	aHEV (avian HEV)	chicken
		*Rocahepevirus ratti*	HEV-C1	rat, shrew, kestrel, falcon, fox
			HEV-C2	ferret, mink
		*Rocahepevirus* *eothenomi*		vole
		*Chiropteranhepevirus*		bat
	*Parahepevirinae*	*Piscihepevirus*		trout, salmon

**Table 2:** Investigation of human hepatitis caused by *Rocahepevirus ratti* virus in different countries.

Continent	Location [Reference]	Screened Patients	Description of Patients Investigated	HEV-C1 RNA Target	HEV-C1 Cases
Asia	Hong Kong 2022 [17]	53	Elderly/Immunocompromised	ORF-1	8/53 (15.1%)
	Hong Kong 2021 [18]	2860	Abnormal liver function (*n* = 2201) Immunocompromised (*n* = 659)	ORF-1	6/2201 (0.27%) 1/659 (0.15%)
	Hong Kong 2018 [19]	518	Persistent Hepatitis (*n* = 52)	RdRp (ORF-1)	1/52 (1.9%)
Africa	D.R.Congo or Gabon * [20]	1	Acute Hepatitis. HEV-IgG (+), HEV-IgM (+/−)	RdRp (ORF-1)	1
Europe	Spain [4]	267	Acute hepatitis unknown etiology (*n* = 169) Acute HEV infection (*n* = 98)	RdRp (ORF-1)	2/169 (1.2%) 1/98 (1.0%)
	Germany [15]	200	HEV suspicion (2000–2020) HEV-IgM (+) or (+/−); HEV RNA (−)	RdRp (ORF-1)	none
	France [21]	224	Individuals at risk of chronic HEV infection	RdRp (ORF-1)	none
	France [22]	1	HEV-IgM (+)	Pan-pathogen metagenomics	1
	Hungary [16]	1439	HEV-IgM (+) (*n* = 162)	Unknown	none

* The country source of infection is not clear. Abbreviations: +: positive; −: negative; +/− borderline; none: none results were found; RdRp: RNA-dependent RNA-polymerase.

**Table 3:** Overview of human infections linked to rat HEV-C1 virus (*Rocahepevirus ratti*) (*N* = 21).

Patient	Underlying Disease	Clinical Outcome	Potential Source of the Infection	Location (Year)	GenBank Acc. Nº	Reference
56-year-old man	Liver transplantation, HBV carrier, immunocompromised	Persistent hepatitis	Rodent droppings	Hong Kong (2017)	MG813927	[19]
71-year-old female	Rheumatoid arthritis, immunocompromised	Acute hepatitis	Unknown	Hong Kong (2017)	MN450851	[18]
67-year-old man	Kidney transplantation, immunocompromised	Persistent hepatitis	Unknown	Hong Kong (2018)	MN450852	[18]
74-year-old man	Kidney transplantation, HBV carrier, immunocompromised	Persistent hepatitis	Unknown	Hong Kong (2018)	MN450853	[18]
81-year-old man	Prostate cancer	Acute hepatitis	Unknown	Hong Kong (2019)	MN450856	[18]
73-year-old man	None, immunocompetent	Acute hepatitis	Unknown	Hong Kong (2019)	MN450857	[18]
67-year-old man	Metastatic cancer	Subclinical	Unknown	Hong Kong (2018)	MN450858	[18]
43-year-old man	HIV infection	Persistent hepatitis	Unknown	Hong Kong (2019)	MN450854	[18]
49-year-old man	None, immunocompetent	Severe acute hepatitis	Trip to Africa	D.R.Congo or Gabon (2019)	MK050105	[20]
62-year-old man	Metastatic oral cancer	Acute hepatitis	Raw food consumption	Spain (2020)	OK082152	[4]
30-year-old man	None, immunocompetent	Acute hepatitis	Cleaning staff	Spain (2018)	OK082153	[4]
54-year-old man	None, immunocompetent	Acute hepatitis	Unknown	Spain (2019)	OK082154	[4]
83-year-old female	Hypertension, tuberculosis	Persistent hepatitis	Unknown	Hong Kong (2020) *	NA *	[17]
18-year-old man	HSCT, immunocompromised	Persistent hepatitis	Unknown	Hong Kong (2020) *	NA *	[17]
61-year-old man	Hypertension	Acute hepatitis	Unknown	Hong Kong (2020) *	NA *	[17]
71-year-old man	Liver transplant, immunocompromised	Persistent hepatitis	Unknown	Hong Kong (2020) *	NA *	[17]
89-year-old female	Pancreatic cancer	Acute hepatitis	Unknown	Hong Kong (2020) *	NA *	[17]
79-year-old man	Hypertension, diabetes	Acute hepatitis	Unknown	Hong Kong (2020) *	NA *	[17]
59-year-old man	Chronic myeloid leukemia	Acute hepatitis	Unknown	Hong Kong (2020) *	NA *	[17]
61-year-old man	Kidney transplantation, immunocompromised	Cirrhosis	Trip to India	France (2017)	OP610066	[22]

* Patients studied between 1 August 2019 and 31 December 2020; Abbreviations: NA: Not Available.

**Table 4:** Global data of animal infections associated with *Rocahepevirus ratti* (*N* = 489).

Continent	Asia	*n*	Europe	*n*	America	*n*	Africa	*n*
	China	159	Germany	42	USA	37	Kenya	1
	Indonesia	117	Czech Republic	20	Canada	21		
	Japan	14	Lithuania	9	Brazil	7		
	Vietnam	5	Romania	9				
			Belgium	8				
			Great Britain	8				
			Hungary	7				
			France	5				
Country RHEV infections			Austria	4				
			Spain	4				
			Switzerland	4				
			Denmark	3				
			Greece	2				
			Netherlands	2				
			Italy	1				
**Total infections**		295		128		65		1

## Data Availability

No new data were created or analyzed in this study. Data sharing is not applicable to this article.
